# The past, current and future epidemiological dynamic of SARS-CoV-2

**DOI:** 10.1093/oxfimm/iqac003

**Published:** 2022-06-20

**Authors:** François Balloux, Cedric Tan, Leo Swadling, Damien Richard, Charlotte Jenner, Mala Maini, Lucy van Dorp

**Affiliations:** UCL Genetics Institute, University College London, London WC1E 6BT, UK; UCL Genetics Institute, University College London, London WC1E 6BT, UK; Genome Institute of Singapore, Agency for Science, Technology and Research (A*STAR), 138672 Singapore, Singapore; Division of Infection and Immunity, University College London, London NW3 2PP, UK; UCL Genetics Institute, University College London, London WC1E 6BT, UK; Division of Infection and Immunity, University College London, London NW3 2PP, UK; UCL Genetics Institute, University College London, London WC1E 6BT, UK; Division of Infection and Immunity, University College London, London NW3 2PP, UK; UCL Genetics Institute, University College London, London WC1E 6BT, UK

**Keywords:** coronaviruses, phylogenetics, host range, specific immunity, epidemic dynamic, endemicity

## Abstract

SARS-CoV-2, the agent of the COVID-19 pandemic, emerged in late 2019 in China, and rapidly spread throughout the world to reach all continents. As the virus expanded in its novel human host, viral lineages diversified through the accumulation of around two mutations a month on average. Different viral lineages have replaced each other since the start of the pandemic, with the most successful Alpha, Delta and Omicron variants of concern (VoCs) sequentially sweeping through the world to reach high global prevalence. Neither Alpha nor Delta was characterized by strong immune escape, with their success coming mainly from their higher transmissibility. Omicron is far more prone to immune evasion and spread primarily due to its increased ability to (re-)infect hosts with prior immunity. As host immunity reaches high levels globally through vaccination and prior infection, the epidemic is expected to transition from a pandemic regime to an endemic one where seasonality and waning host immunization are anticipated to become the primary forces shaping future SARS-CoV-2 lineage dynamics. In this review, we consider a body of evidence on the origins, host tropism, epidemiology, genomic and immunogenetic evolution of SARS-CoV-2 including an assessment of other coronaviruses infecting humans. Considering what is known so far, we conclude by delineating scenarios for the future dynamic of SARS-CoV-2, ranging from the good—circulation of a fifth endemic ‘common cold’ coronavirus of potentially low virulence, the bad—a situation roughly comparable with seasonal flu, and the ugly—extensive diversification into serotypes with long-term high-level endemicity.

## INTRODUCTION

On New Year’s Eve of 2019, a cluster of cases of pneumonia was reported in Wuhan, China. The causative agent was identified as a novel coronavirus and the first genome sequence was made available to the scientific community by mid-January 2020 [1]. Soon thereafter, an additional five complete genomes collected from patients infected during the early stages of the outbreak were reported. Sequences were near-identical, a result in line with a recent host jump into humans from a single source [2]. The newly identified coronavirus, initially called 2019-nCoV, was renamed SARS-CoV-2 due to its relatedness to SARS-CoV-1, the agent of the 2003 SARS outbreak. These early efforts were followed by a remarkable and unprecedented undertaking by the international scientific community to sequence large numbers of SARS-CoV-2 genomes. Over 500 genomes from 39 countries and 9 continents were available by the time the WHO declared the COVID-19 pandemic on 11 March 2020, and the milestone of 1 million SARS-CoV-2 genomes was reached a year later in March 2021 [3]. The international sequencing effort intensified as the pandemic progressed and by March 2022 over 10 million SARS-CoV-2 genomes had been deposited on the GISAID database [4, 5].

Phylogenetic analyses point to a Most Recent Common Ancestor (MRCA) of all sequenced SARS-CoV-2 dating back to late 2019 [6, 7]. When the WHO announced that the outbreak of pneumonia in Wuhan was caused by a novel coronavirus on 9 January 2020, 59 cases had been reported in Wuhan, China. While some of the earliest cases have epidemiological links to the Huanan Seafood Wholesale Market [8], the lack of direct isolates from animals at the market [9] leaves the proximal origin and early stages of the COVID-19 pandemic timeline largely unclear. The first confirmed cases outside China were identified in Thailand and Japan on 20 January, and a day later, a Washington state resident who had returned from Wuhan on 15 January tested positive [10]. The first confirmed cases in Europe were reported in France on 24 January 2020 [11]. Athough it is suspected SARS-CoV-2 was present in Europe earlier, in particular with some evidence the virus may have been circulating in Northern Italy already in December 2019 [12].

Genome sequencing can shed only limited light on the early timeline of the pandemic due to the small number of genomes available from that period. Athough from March 2020 onwards, the global spread and genetic evolution of SARS-CoV-2 can be exquisitely documented. Over the first half of 2020, the global population of SARS-CoV-2 remained largely unstructured geographically, with the entire viral genetic diversity distributed in most regions of the world [6, 13], with the notable exception of China, which rapidly contained the initial outbreak. As different countries implemented travel bans and other mitigations measures, the global SARS-CoV-2 population became more geographically structured with different viral lineages attaining high prevalence locally from mid-to-late 2020 onwards [14, 15].

From the start of the pandemic, multiple lineages emerged while others went extinct, thereby keeping the genetic diversity of the SARS-CoV-2 population in circulation at a largely constant level during the first 2 years. Until the near-simultaneous characterization of four variants of concern (VoCs) towards the end of 2020 and the Omicron VoC in late 2021, this lineage-replacement dynamic remained mostly unnoticed with the exception of the emergence and global spread of the B.1 lineage carrying the D614G mutation that swept the world during the early stages of the pandemic [16, 17]. The more transmissible Alpha VoC, which emerged in the UK, spread globally [18, 19]. However, before it reached worldwide prevalence, it was replaced by the even more transmissible Delta VoC and daughter lineages, first identified in India, in 2021 [20, 21]. Delta was subsequently displaced by the Omicron VoC, identified in late November 2021 in South Africa [22], and which rapidly established global prevalence. Other designated VoCs of the pandemic include Beta, first detected in South Africa [23], and Gamma, first identified in cases linked to Brazil [24]. The emergence of VoCs represented a sharp turn in the evolutionary picture of SARS-CoV-2 and prompted the need for the early definition (variants under investigation/monitoring), detection and monitoring of lineages that pose an increased risk to global health.

With the pandemic in its third year, it is becoming increasingly urgent to understand the long-term evolutionary dynamics of SARS-CoV-2. Here, we first overview key aspects of SARS-CoV-2 genetic and immunogenetic evolution and draw parallels with what is known for other coronaviruses infecting humans. We conclude the review by delineating possible scenarios for the future.

## AN OVERVIEW OF CORONAVIRUSES

**Figure 1:**
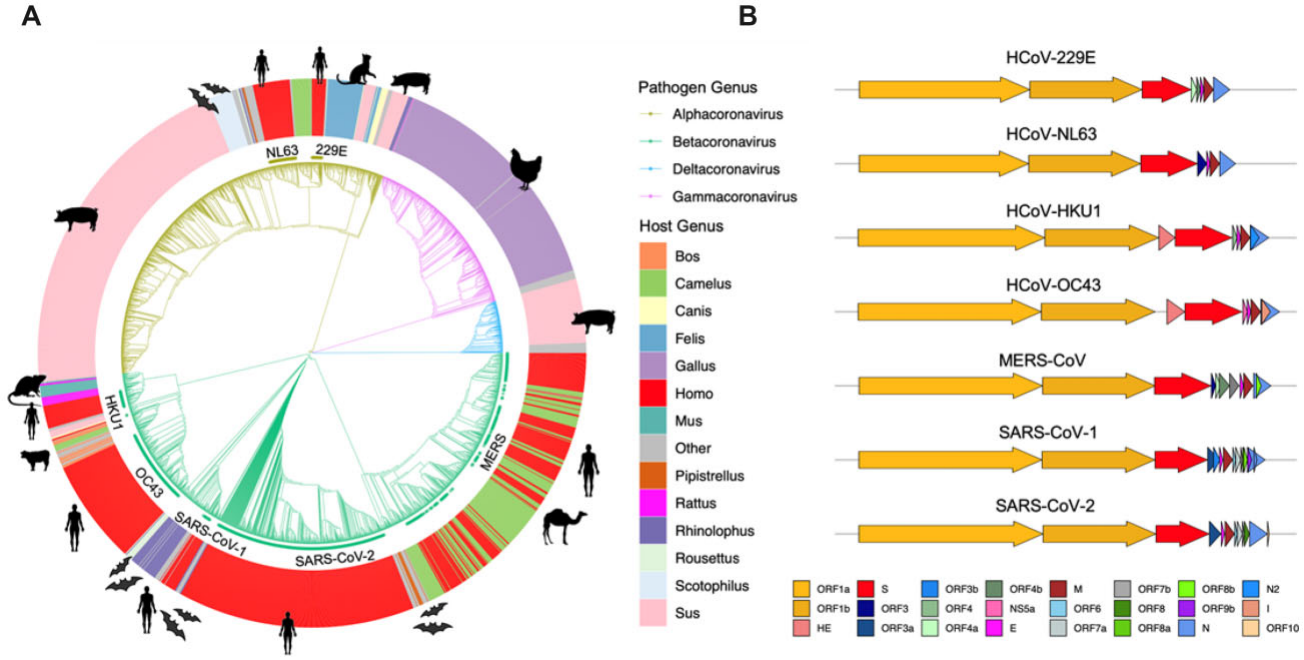
Overview of the *Orthocoronavirinae*. *Notes*: (**A**) Phylogenetic representation of 2529 coronavirus genomes. The branch colour indicates the assignment into the four genera (*Alpha*-, *Beta*-, *Gamma*- and *Deltacoronavirus*). The coloured outer circle and icons provide the host the strains were isolated from. Each of the seven human-associated coronaviruses (HCoVs) are highlighted on the inner ring. (**B**) Genome structure of major human associated coronaviruses. Gene annotations are provided for the coding RNA sequences of seven representative human-associated coronaviruses (HCoV-229E NC_002645.1; HCoV-NL63 JX504050.1; HCoV-HKU1 NC_006577.2; HCoV-OC43 KX344031.1; MERS-CoV NC_019843.3; SARS-CoV-1 NC_004718.3; and SARS-CoV-2 NC_045512.2). Gene annotations are provided as per the colour legend at bottom, aligned to the coronavirus spike protein. Figure made using the R package gggenes (https://cran.r-project.org/web/packages/gggenes).

Coronaviruses belong to the viral family Coronaviridae, which comprises three subfamilies: Orthocoronavirinae, Pitovirinae and Letovirinae. The Orthocoronavirinae subfamily comprises the four genera: *Alpha-*, *Beta-*, *Gamma-* and *Deltacoronavirus* [25]. SARS-CoV-2 falls within the *Sarbecovirus* subgenus in the genus *Betacoronavirus*, together with SARS-CoV-1, the agent of the SARS 2002–04 epidemic, and related lineages circulating in populations of horseshoe bats (*Rhinolophus* sp.). *Betacoronavirus* also include MERS-CoV and two seasonal human endemic coronaviruses: HCoV-OC43 and HCoV-HKU1 (Fig. 1A). Lineages within *Gammacoronavirus* are restricted to birds but Alpha- and Deltacoronaviruses infect mammals [26]. Deltacoronaviruses have been primarily isolated from domestic pigs, but Alphacoronaviruses infect a broad range of mammals, including humans, with the genus comprising the two other seasonal human endemic coronaviruses HCoV-229E and HCoV-NL63 (Fig. 1A).

Coronaviruses are enveloped viruses with a single-strand, positive-sense RNA genome which, at ∼26–32 kilobases, is among the largest known genomes for RNA viruses (Fig. 1B). The basic organization of the genome is conserved across members of the *Orthocoronavirinae*. The bulk of the genome (around two-thirds) extending from the 5′-end comprises the replicase open-reading frame (ORF) 1a/b which encode two polyproteins which are cleaved into a suite of non-structural proteins (NSPs; ORF1a: NSP1-11, ORF1b: NSP12-16) involved in proteolytic processing, transcription and genome replication [27, 28]. These include the NSP12 RNA-dependent RNA polymerase that is essential for RNA synthesis and associated exoribonuclease NSP14 that exhibits proof-reading activity [29]. The remaining third of the genome encodes the structural proteins in 5′- to 3′-order; the surface or spike glycoprotein (S), the membrane glycoprotein (M), the envelope (E) and the nucleocapsid (N).

Of all ORFs identified in the *Orthocoronavirinae*, only ORF1ab, S, M and N can be considered homologues in >99% of sequenced coronavirus genomes (i.e. ‘core’ genes). Additionally, only ∼43% of the average coronavirus genome can be meaningfully aligned at the nucleotide level [30]. Some Betacoronaviruses, most notably HCoV-HKU1 and HCoV-OC43, harbour an additional hemagglutinin-esterase (HE) glycoprotein which can act as a secondary viral attachment protein [31]. Additionally, coronavirus genomes typically harbour a variable complement of accessory proteins depending on the genus, or even the individual species [32] (Fig. 1B).

Viral characterization has particularly focused on the spike glycoprotein, whose club-shaped surface projections from the lipid bilayer envelope give coronavirus virions their appearance [33]—reminiscent of the sun’s corona or halo during an eclipse [34]. The spike protein plays a key role in viral entry in the host cell, mediating host receptor recognition, cell attachment and membrane fusion [35, 36]. Notably, the host receptors recognized and used by the spike for viral entry vary between different coronavirus species. SARS-CoV-2 binds to the angiotensin-converting enzyme 2 (ACE2), as does SARS-CoV-1 and HCoV-NL63, whereas other species within the same genus (including MERS-CoV) recognize a cell-surface serine peptidase, dipeptidyl peptidase 4 (DDP4), and others bind with 9-*O*-acetylated sialic acid (HCoV-OC43 and HCoV-HKU1) [32, 37–39]. Finally, HCoV-229E uses the human aminopeptidase N (hAPN) receptor [40]. All four host receptors are expressed in a wide range of human cell types, including those in the respiratory and gastrointestinal tracts [29–31], which may explain the broad tissue tropism and hence the diverse symptoms and transmission routes of coronavirus infections in humans [35].

The spike protein comprises two subunits, known as S1 and S2, with the former including important regions such as the N-terminal domain (NTD) and receptor binding domain (RBD) that directly binds host receptors during viral entry [38, 41]. The S2 domain additionally supports the fusion and eventual entry of the virus into host cells. Cell entry by SARS-CoV-2 is dependent on the activation of the spike protein by proteases. TMPRSS2 and cathepsin L are the two main proteases activating the spike protein [42]. TMPRSS2 is co-expressed together with ACE2 on the surface of most cells of the gastrointestinal, respiratory and urogenital epithelium [43]. If a virus–ACE2 complex fails to bind TMPRSS2, it can still enter cells through endocytosis and S2 is then activated by cathepsins [42]. Given its critical role in receptor binding, membrane fusion and immunogenicity, the spike protein is subject to natural selection for enhanced infectivity and antigenicity [44]. The S1 and S2 regions as well as the S1/S2 junction are the only targets of neutralizing antibodies (antibodies precluding pathogen entry into host cells). Due to this property, the spike protein was selected as the sole source of antigens in the majority of COVID-19 vaccines developed so far, including those based on mRNA (Pfizer/BioNTech/Moderna) and adenovirus recombinant technologies (Oxford/AstraZeneca/Sputnik V/Johnson and Johnson).

Coronavirus accessory proteins are more poorly characterized. While these proteins are generally not considered essential for viral replication [45], they play a role in pathogenesis, host interactions and transmissibility [46, 47]. ORF3a, the largest accessory protein in SARS-CoV-2, has been shown to interact strongly with the host immune system, activating the NLRP3 inflammasome and contributing to the generation of a cytokine storm [48]. Its functional role in cell apoptosis has been documented for both SARS-CoV-1 [49] and SARS-CoV-2 [50]. Consistently, in a SARS-CoV-1 animal model, deletion of ORF3a was shown to reduce viral replication [51]. The accessory proteins ORF6, ORF7a and ORF8 act as interferon antagonists in SARS-CoV-2 [46]. Of these, SARS-CoV-2 ORF8 is particularly interesting in that its presence as an intact gene (rather than fragmented into ORF8a/b) is unique among human-associated coronaviruses, with ORF8 being identified as highly immunogenic among the accessory genes [52]. A large deletion in ORF8 (Δ382) in SARS-CoV-2 circulating in a cohort of patients in Singapore in early 2020 was identified as being potentially associated with milder disease [53], further reinforcing its possible role in pathogenesis. While coronavirus accessory proteins have been studied most closely in SARS-CoV-2, many uncertainties remain about the exact role of different ORFs. For instance, to date no function has been associated to ORF10 and it has been questioned whether it should be considered a gene [54].

## THE EVOLVING HOST RANGE OF SARS-CoV-2

Despite remarkable global genome sequencing and characterization efforts, the proximal phylogenetic origin of SARS-CoV-2 and its mode of introduction into human circulation remain unclear. There is no evidence for SARS-CoV-2 having been ‘engineered’ in a lab. Conversely, the escape of a strain from a lab or an accidental contamination during field work cannot formally be ruled out at this stage. However, a zoonotic spillover event in nature is considered as the most plausible scenario in the scientific community [2, 55–58]. A diversity of viruses closely related to SARS-CoV-2 have been isolated from multiple species of horseshoe bats (*Rhinolophus* sp.) sampled across East and Southeast Asia [2, 56–61]. Thus far, RaTG13 isolated from *Rhinolophusaffinis* in Yunnan in 2013 shares the highest whole-genome sequence identity with SARS-CoV-2 at 96.2% [2], followed by RpYN06 from *Rhinolophuspusillus* at 94.5% [57]. However, identity along the genome is highly variable. For example, phylogenetic analysis identified RmYN02 (*Rhinolophusmalayanus*; 93.3% identity) as the closest relative over the ORF1ab region (97.2%) [58]. The lower whole-genome sequence identity relative to that for RaTG13 is largely due to the sequences in the spike (71.9% sequence identity) [58]. However, at the region critical for host receptor binding—the RBD—RaTG13 has a low genetic similarity to SARS-CoV-2.

**Figure 2:**
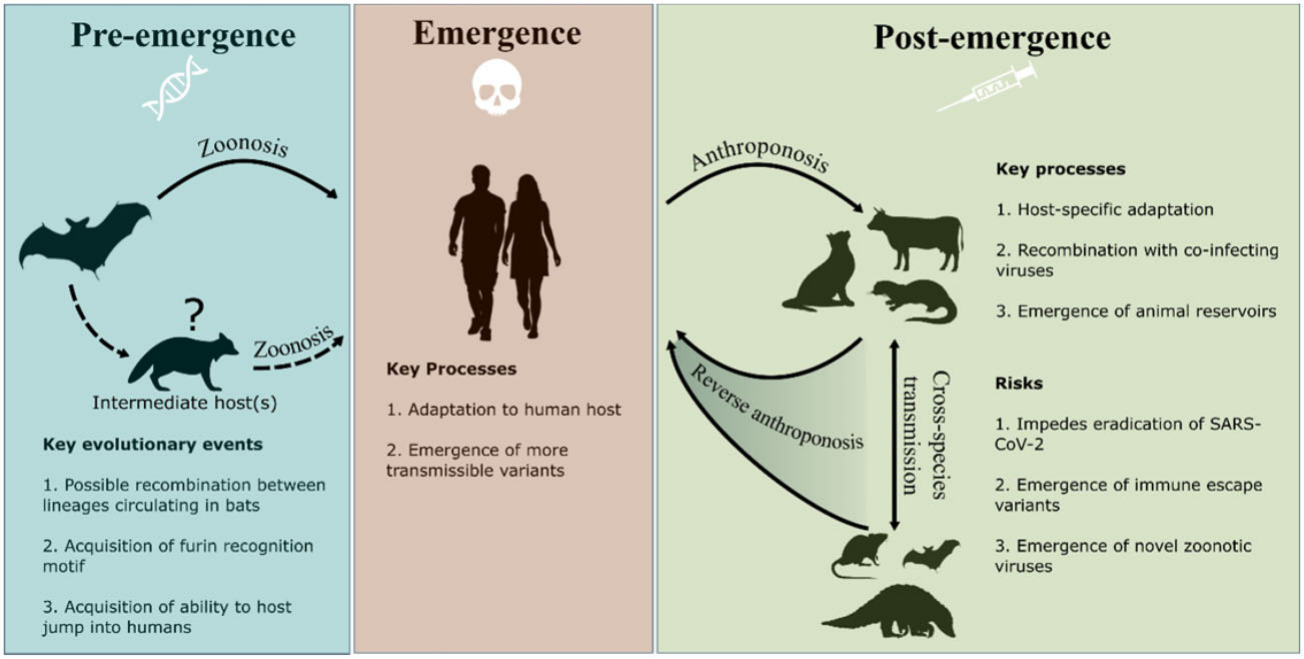
SARS-CoV-2 host range evolution before, during and after the COVID-19 pandemic. *Notes*: The box on the left summarizes possible scenarios predating the host jump into humans, followed by key processes during the establishment of human-to-human transmission (central box). The box at the right displays the processes and risks associated with secondary spillovers from humans to wild and domestic animals. Icon choice is emblematic, sourced from flaticon.com.

Other bat coronaviruses, most notably those recently recovered from *R. malayanus*, *R.marshalli* and *R. pusillus* in Northern Laos, Indonesia, harbour RBD motifs far closer to SARS-CoV-2 and have been demonstrated to efficiently bind to human ACE2 [59]. The genetic similarity between RaTG13 and SARS-CoV-2 is largely comparable to that of the viral lineages most closely related to SARS-CoV-1 found in horseshoe bats (*Rhinolophus* spp.) [62, 63]. As such, it may be argued that the progenitor of SARS-CoV-1 has never been identified. Although in contrast to the situation for SARS-CoV-2, viral strains with near-perfect whole-genome sequence identity have been isolated from captive Himalayan palm civets (*Paguma larvata*) and a raccoon dog (*Nyctereutes procyonoides*) [64]. These observations led to the hypothesis that a carnivore may have acted as an intermediate host in the jump of SARS-CoV-1 into humans [65]. Early in the COVID-19 pandemic, there were suggestions that pangolins (*Manis javanica*) could have acted as an intermediate host for SARS-CoV-2 [66]. However, current evidence does not indicate that an established reservoir outside of bats may have been required for the host jump into humans [67] (Fig. 2).

Studies of the proximal origins of SARS-CoV-2 have focused on the Arginine-Arginine-Alanine-Arginine (RRAR) furin recognition motif at the S1/S2 spike subunits junction. Pre-activation of the SARS-CoV-2 spike by host proprotein convertase furin at this motif is crucial for cellular entry [68], viral replication and pathogenesis [69]. The presence of the RRAR motif in SARS-CoV-2 and its absence in close relatives has been suggested as a sign of bioengineering. However, while close relatives such as RmYN02 lack the exact furin recognition motif, they share a genomic region homologous to that in SARS-CoV-2 (positions 23603–23615) within the spike protein [70]. This observation suggests that recombination with a yet unsampled coronavirus may have played a role in the emergence of the furin recognition motif in SARS-CoV-2. Further, furin recognition motifs (RXXR) have emerged independently multiple times in the evolution of *Betacoronaviruses* [71], with MERS-CoV, HCoV-OC43 and HCoV-HKU1 also carrying furin cleavage sites. Hence, the natural emergence of the RRAR motif in SARS-CoV-2 through recombination and/or other evolutionary processes (point mutations and indels) represents a parsimonious explanation (Fig. 2).

As with other coronaviruses, SARS-CoV-2 can infect and transmit efficiently within different populations of mammals (Fig. 1A). This is evidenced by the plethora of studies that have probed the host tropism of SARS-CoV-2, *in vitro* (cell lines), *in vivo* (live inoculation) and through wildlife surveillance (Table 1). Human-to-animal spillover (i.e. anthroponosis) of SARS-CoV-2 into multiple wild, captive and domestic mammalian species has been observed repeatedly and is particularly well-documented in zoo animals [72], farmed mink [73, 74] and wild white-tailed deer [75, 76] (Table 1). Notably, evidence for natural or experimental infection may not necessarily entail efficient animal–animal transmission. For example, porcine cell lines are permissive to infection [77, 78] but studies have failed to experimentally infect pigs *in vivo* [78, 79]. Further, while animal hosts such as dogs and cattle have been shown to be susceptible to infection, transmission is poor or non-existent [80, 81], indicating that SARS-CoV-2 is not yet adapted for efficient transmission in these species. The broad host tropism of SARS-CoV-2 may be in part due to the usage of the ACE2 receptors for viral entry. ACE2 is fairly conserved across vertebrates [82], which entails that the accumulation of only few mutations may be required to evolve efficient binding to receptors in a novel host species. To exploit this, several studies have suggested that bioinformatic screening of key residues on animal ACE2 that govern binding affinity may be useful in assessing potential animal reservoirs [82–85].

**Table 1:** Reports of susceptibility of different animal hosts to SARS-CoV-2

Host	Species	In vitro susceptibility	In vivo susceptibility	Transmission	Detected in wild	Anthroponosis	Reverse anthroponosis
White-tailed deer	*Odocoileus virginianus*	Yes	Yes [86, 87]	Yes [86, 87]	Yes [75]	Yes [76, 88]	Probable [89]
American mink/ European mink	*Neogale vison*/ *Mustela lutreola*	Yes	Yes	Yes [73, 74]	Yes [90]	Yes [74]	Yes [74]
Other mustelids	*Mustela putorius furo* *Martes martes* *Meles meles*	Yes	Yes [91]	Yes [79, 91–93]	Yes [94]	Yes [94]	
Domestic cats	*Felis catus*	Yes [77]	Yes [95, 96]	Yes [95, 97]		Yes [98, 99]	
Dogs	*Canis lupus familiaris*	Yes	Yes [95, 96]	No [95]		Yes [81, 99]	
Big cats	*Panthera* spp. *Puma concolor*	Yes^a^	Yes [72]			Yes [72]	
Spotted hyenas	*Crocuta crocuta*	Yes^a^	Yes^a^			Yes^a^	
Bearcat	*Arctictis binturong*	Yes^a^	Yes^a^			Yes^a^	
Fishing cat	*Prionailurus viverrinus*	Yes^a^	Yes^a^			Yes^a^	
Canada Lynx	*Lynx canadensis*	Yes^a^	Yes^a^			Yes^a^	
Coati	*Nasua nasua*	Yes^a^	Yes^a^			Yes^a^	
Cattle	*Bos Taurus*	Yes	Yes [80]	No [80]			
Pigs	*Sus scrofa*	Yes [77, 78]	No [78, 79]				
Chickens	*Gallus gallus*		No [79]				
Fruit bats	*Rousettus aegyptiacus*	Yes	Yes [79]				
Horseshoe bats	*Rhinolophus sinicus*	Yes [77, 100, 101]					
Vesper bats	*Pipistrellus abramus*	Low [101]					
Rats/mice	*Rattus norvegicus* *Mus musculus*	Variants only [102]	Variants only [102]	No [102]			
Hamsters	*Mesocricetus auratus*	Yes	Yes [103]			Yes [104]	Yes [104]
Non-human primates	*Macaca mulatta* *Macaca fascicularis* *Chlorocebus aethiops*	Yes	Yes [77, 103]				

a https://www.aphis.usda.gov/aphis/dashboards/tableau/sars-dashboard; green: confirmed positive; red: confirmed negative; orange: unconfirmed; yellow: some variants only.

The host range of SARS-CoV-2 is likely dynamic and the virus may further expand its host repertoire in the future. For example, mice and rats were not susceptible to the first strains of SARS-CoV-2 circulating in humans [102]. However, following the global spread of the Alpha VoC, which carries the N501Y mutation facilitating infection in those species, they have become susceptible hosts [105]. Given the broad host tropism of SARS-CoV-2, there is a risk that viral mutations may emerge while circulating in novel animal host species following anthroponotic spillover. This entails a further risk that the transmission of animal-adapted SARS-CoV-2 back into humans (i.e. reverse anthroponosis) may alter the evolutionary trajectory of the virus, potentially leading to the emergence of more transmissible or immune-escape variants (Fig. 2). There is evidence for the emergence of host-adaptive mutations in SARS-CoV-2 strains circulating in farmed minks and wild white-tailed deer that have arisen recurrently and have reached a high frequency [106, 107]. For example, spike mutations, Y453F and F486L, are good candidates for adaptation in mink in addition to dampening human humoral and T cell-mediated immunity [108–111]. Similarly, following introduction in a research colony of cats [95], spike mutation H655Y rapidly reached fixation; a substitution that has been associated with immune escape in humans [112]. Reverse-anthroponotic transmission of SARS-CoV-2 has been documented for farmed minks [74, 106], white-tailed deer [89] and pet-shop hamsters [104] and it is to be expected that SARS-CoV-2 can also jump from other animal species back into humans. Putatively, mink-adaptive mutations are currently only maintained at a low frequency in human-associated SARS-CoV-2, suggesting that they do not confer a significant advantage in human circulation [106, 111]. As yet, the circulation of SARS-CoV-2 in mink and deer has not resulted in a significant alteration in extant genomic diversity [106].

The emergence of reservoirs in wildlife is of further concern since it is more difficult to assess and control anthroponotic or reverse-anthroponotic movement of SARS-CoV-2 between humans and wild animal populations than domestic species. Such reservoirs may emerge due to direct anthroponotic transmission from humans living in close proximity to wild animals, or indirect transmission via domesticated animals or contaminated water sources. SARS-CoV-2 has already established itself in wild white-tailed deer populations with a detected seroprevalence of 7–67% across different states in the USA [75, 76]. Further, there have been reports of deer-associated viral sequences that are considerably divergent from the human-associated sequences sampled within the same geographic region and time period [89, 113]. Given the broad host tropism of SARS-CoV-2, the emergence of other wild or farmed animal reservoirs remains a possibility. This may be particularly problematic in animals that already harbour a vast diversity of coronaviruses such as bats [56, 114, 115], since the propensity for recombination in coronaviruses [116] might lead to the emergence of novel recombinant viruses with the ability to transmit efficiently in humans (Fig. 2).

## SARS-CoV-2 IN THE CONTEXT OF OTHER HUMAN CORONAVIRUSES

**Figure 3:**
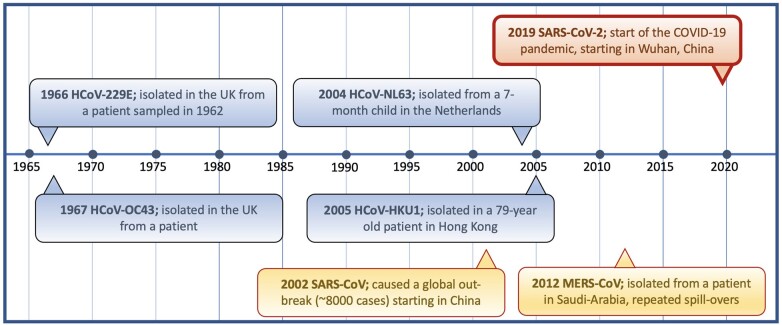
Timeline of the age of first observation for the seven HCoVs. *Notes*: Endemic coronaviruses are shown in blue, epidemic coronaviruses in yellow and pandemic SARS-CoV-2 is represented in red. Additionally, in the 1960s, several strains including B814, 692 and HCoV-OC16, -OC37 and -OC48 were observed, but lost for follow-up studies. It is unclear whether these strains may have been later identified or represent yet uncharacterized HCoVs. The figure is informed by the following sources [2, 117, 118, 124–126].

Prior to the emergence of SARS-CoV-2, six other coronaviruses were known to commonly infect humans (Fig. 1). HCoVs were first observed in the 1960s in samples at the Common Cold Unit in Salisbury, England [117] (Fig. 3). However, they were not considered highly pathogenic until the outbreak of SARS-CoV-1 in 2002 [118]. SARS-CoV-1 affected at least 8000 individuals causing severe respiratory disease with a case fatality rate of ∼10% [119]. Approximately 10 years later, MERS-CoV was identified in Saudi Arabia, thought to have also originated in bats [120] (Fig. 3). MERS-CoV also causes severe-to-fatal respiratory disease in humans and since its discovery over 2500 MERS-CoV cases have been reported with >850 associated deaths [121]. MERS-CoV is not well adapted to transmission in humans and is believed to spread through zoonotic reservoirs (mainly dromedary camels) with frequent ongoing spillovers into susceptible human populations [122]. MERS-CoV has been circulating in dromedary camels for decades as evidenced by the presence of MERS neutralizing antibodies in samples from East Africa dating back to 1983 [123].

The four endemic respiratory seasonal coronaviruses (HCoV-229E, -OC43, -NL63 and -HKU1) circulate in all age groups with the first exposure generally happening in childhood. HCoVs tend to cause mild self-limiting upper respiratory or gastrointestinal disease in the immunocompetent accounting for ∼10–30% of common cold cases [127–129]. HCoVs tend to induce only relatively short-term protection against reinfection, which occurs repeatedly throughout life [130, 131]. This is likely due to fairly rapid waning of post-infection antibody titres to coronaviruses but there is also evidence that HCoV-OC43 and -229E undergo constant adaptive evolution to escape host immune recognition [132]. Both HCoVs tend to constantly acquire mutations in the S1 region of the spike protein and their genetic diversity over three decades falls into ladder-like phylogenetic trees compatible with antigenic drift, similar to those observed for seasonal flu (influenza A) [132]. Moreover, it has been demonstrated that historic sera able to potently neutralize virions pseudotyped with contemporary HCoV-229E spike proteins had little to no activity against spike proteins from HCoV-229E strains isolated 8–17 years later [133]. In the same study, modern adult sera were found to neutralize spike proteins from a variety of historical viruses, whereas modern sera from children best neutralized spike proteins from recent viruses that would have circulated in their lifetime [133]. These patterns provide evidence for antigenic evolution of the CoV spike protein in HCoV-229E, particularly in the spike RBD permitting the escape of neutralization by polyclonal sera within one or two decades [133]. No consistent evidence for antigenic drift has been found to date for HCoV-NL63 and -HKU1, but this may simply reflect the lack of sufficient longitudinal sequencing data to identify adaptive evolution [134].

Estimates for the age of emergence of the four endemic HCoVs remain under debate, though it is widely accepted that they were in circulation a good deal prior to first observations. For example, while HCoV-NL63 was first identified in 2004 (Fig. 3), the earliest available genome was later generated from a sample collected in 1983 [135] and the inferred MRCA of HCoV-NL63 strains currently in circulation has been estimated to date back to the ∼1920s [136]. HCoV-229E and -NL63 likely evolved in bats [137, 138], with camelids having possibly acted as an intermediatory for HCoV-229E transmission to humans [139]; a scenario similar to that of MERS-CoV. Both HCoV-OC43 and -HKU1 are thought to have originated in rodents and there is debate over whether cattle, pigs or other animals may have acted as an intermediate host for HCoV-OC43, with bovine coronavirus suggested as a possible ancestor [140]. It has also been hypothesized that the Russian flu pandemic (1889–1890) may have been caused by HCoV-OC43 [141, 142]. This hypothesis is primarily based on the list of respiratory, gastrointestinal and neurological symptoms including loss of taste and smell recorded at the time, which are more reminiscent of COVID-19 than influenza infections. Early phylogenetic analysis of the HCoV-OC43 spike protein sequence pointed to a time to the MRCA (tMRCA) compatible with a HCoV-OC43 host jump into humans in the late 19th century [140]. However, this estimated date does not seem supported by analyses of additional genomes, which point to a more recent common ancestor for HCoV-OC43 currently circulating in humans [134, 143].

Additional coronavirus spillovers into humans have been documented for species outside of the seven generally categorized as ‘human coronaviruses’. There is evidence of multiple separate cases of cattle strains being transmitted to humans. For instance, two samples of human enteric coronavirus 4408 strains have been documented, collected in 1988 and 2009 [135]. Further, there are clinical, epidemiological and serological observations that bovine coronaviruses can infect human subjects causing diarrhoea [144] and a recent study indicated that bovine coronavirus can be carried in human nasal mucosa after exposure to virus-shedding calves [145]. Coronavirus RNA identified as a novel canine–feline recombinant alphacoronavirus (since named CCoV-HuPn-2018) has been detected in nasopharyngeal swab samples from eight patients during 2017–18 in Sarawak, Malaysia [146]. Additionally, isolates with high identity to feline enteric coronavirus (*Alphacoronavirus*) were detected in three nasal swab samples collected in Arkansas in 2010, from patients exhibiting acute respiratory symptoms. One of these samples contained a 400-base pair fragment in the S region, which revealed an OC43-like spike protein, suggestive of feline coronavirus and HCoV-OC43 coinfection [147]. Porcine deltacoronavirus strains were also recently identified in blood plasma samples from three children in Haiti with acute undifferentiated febrile illness [148]. Prior to this, documented human coronavirus infections had been limited to *Alpha*- and *Betacoronavirus* strains; therefore, this example provides the first known case of *Deltacoronavirus* adaptation to human transmission [148].

These recently documented spillover events point to a wider diversity of coronaviruses circulating in wild and domestic animal populations being able to infect humans than previously recognized. Together with the emergence of three pandemic/epidemic human coronaviruses in the last two decades (SARS-CoV-1, SARS-CoV-2 and MERS-CoV), this observation illustrates the relative ease with which coronaviruses can jump into humans and sometimes subsequently adapt and spread. Coronaviruses infecting humans cover the entire epidemiological spectrum, ranging from rare to common spillover events, outbreaks, epidemics, a pandemic to a global endemic quasi-equilibrium state achieved by the four seasonal HCoVs.

## GENETIC EVOLUTION OF SARS-CoV-2

**Figure 4:**
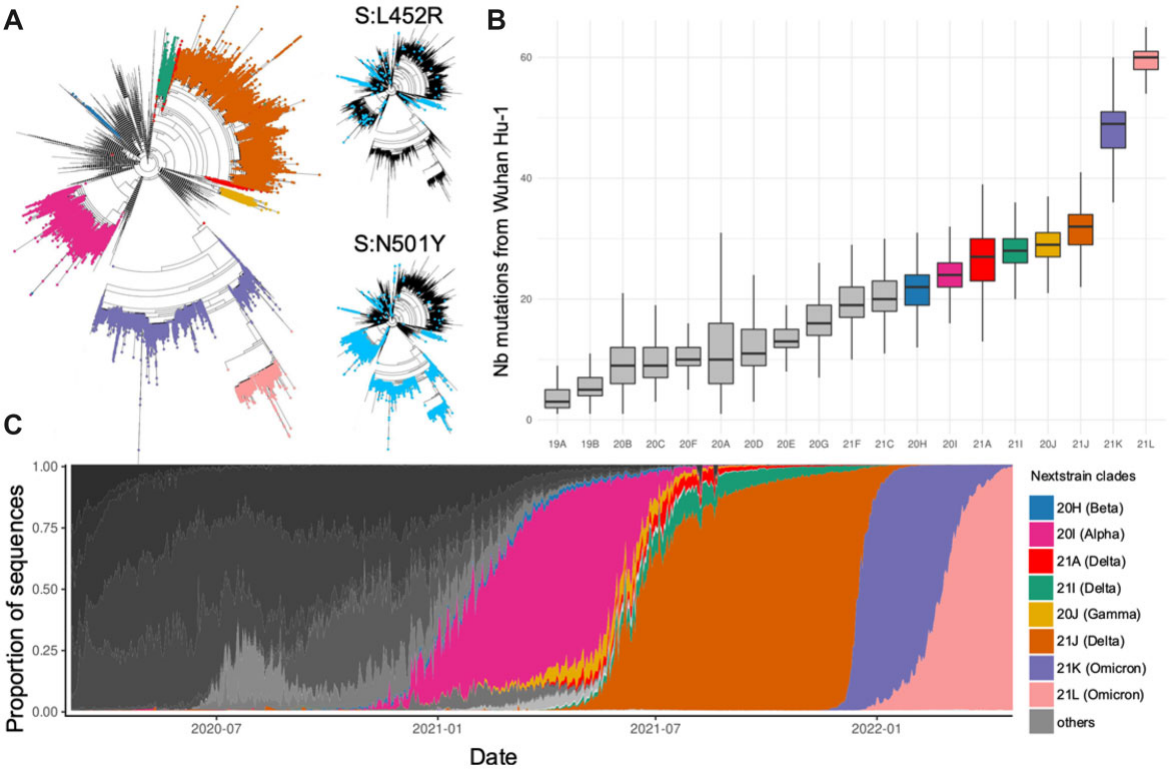
Lineage dynamic of the SARS-CoV-2 population. *Notes*: (**A**) Phylogenetic tree of ∼200,000 SARS-CoV-2 strains coloured by their Nextstrain clade assignment with the inset providing the presence of the Spike L452R and N501Y mutations highlighting the recurrence of independent emergence of mutations in multiple lineages. (**B**) Distributions of distances (in number of mutations) from the root of the tree (Wuhan-Hu-1) of Nextstrain clades (ordered by emergence). (**C**) Daily prevalence of each NextStrain clade estimated as the proportion of uploads to the GISAID genome database highlighting the dynamic of lineage replacement in SARS-CoV-2. Only Nextrain clades corresponding to VoC lineages were attributed a color, others were represented in grey.

The genetic diversity of SARS-CoV-2 was initially very low and mutations accumulated steadily over the first 2 years of the pandemic at a rate of approximately 25–30 mutations per lineage, per year (Fig. 4A) [3]. Interestingly the same mutations can often be observed at a low frequency in many different lineages (Fig. 4A insets). As mutations accumulated over time, this led to more recently emerging lineages being a larger number of mutations away from the hypothetical ancestor of all SARS-CoV-2 strains circulating in humans (Fig. 4B). Even before the emergence of the Alpha, Delta and Omicron VoCs that successively swept the world, the dynamic of the SARS-CoV-2 population was characterized by a birth and death dynamic of lineages replacing each other (Fig. 4C). Interestingly, despite all three variants having achieved global prevalence, they do not derive from each other (Fig. 4A). Both the Delta and then the Alpha variant are descendants of early pandemic lineages that had been displaced globally first by Alpha and then Delta. It remains to be seen whether this pattern will persist, or whether successful variants of the future will derive from each other as is typical for seasonal flu or HCoVs.

Direct competition between lineages can only occur when two lineages simultaneously infect the same host leading to only one lineage being successfully transmitted. Even when SARS-CoV-2 is at high prevalence, such mixed infections are expected to be rare. Thus, the dynamic of lineage replacement is likely primarily fuelled by the emergence of new strains that are more transmissible and/or more able to (re-)infect hosts immunized through vaccination or prior infection. Additionally, fitness decay of lineages in circulation through the accumulation of slightly deleterious mutations (Muller’s ratchet [149]) may also play a role in lineage extinction [150, 151].

The mutation rate of SARS-CoV-2 is fairly unremarkable among RNA viruses [152–154] despite the proof-reading activity of its replication mechanism [155]. While genetic diversity of the SARS-CoV-2 population in circulation remains low 2 years into the pandemic (Fig. 4A and B), mutations can at this stage be found at every single base of the SARS-CoV-2 genome [4, 5]. The majority of these mutations have independently emerged many times in unrelated lineages [6, 15] (Fig. 4A). The bulk of the segregating diversity in SARS-CoV-2 is expected to be adaptively neutral or slightly deleterious. However, early selective sweeps of mutations essential for transmissibility in humans may have been missed [156]. Indeed, the earliest available SARS-CoV-2 genomes were sampled in late December 2019, by which time the virus had probably been in human circulation for several weeks [6].

Host antiviral defence mechanisms inducing mutations at specific sites of the viral genome [15, 157, 158] represent an additional selective force shaping the genetic diversity segregating in the SARS-CoV-2 population. Host-mediated editing of SARS-CoV-2 includes the activity of proteins form the APOlipoprotein B Editing Complex (APOBEC) and the Reactive Oxygen Species and Adenosine Deaminase Acting on RNA (ADAR), all suspected to introduce mutations in a nucleotide and sequence-context specific manner. For instance, the mutation spectrum of SARS-CoV-2 is largely dominated by C-to-U and G-to-U substitutions [15, 159]. These processes, coupled with ongoing selective reduction of CpGs [157, 159], create complex mutational dynamics [159, 160]. Host antiviral defence likely provides a potent selective pressure following spillover to a new host. As such, with the establishment of widespread human-to-human transmission, the rate of evolution in SARS-CoV-2 might slow down, with increasing fixation of advantageous mutations and depletion of sites susceptible to host-induced mutagenesis.

The accumulation of diversity in SARS-CoV-2 motivated the development of classification schemes to designate distinct lineages (Fig. 4A and C). Among the two most widely used SARS-CoV-2 classification schemes are the Nextstrain clade naming system, which provides an up-to-date nomenclature based on phylogeny, VoC status and global and regional frequency [161] and the PANGO nomenclature scheme which relies on the phylogeny and carriage of diagnostic mutations [162]. The latter classification includes many more sub-divisions (numbered labels). While such schemes are essential tools in scientific discourse, any taxonomic delineation into lineages is bound to remain somewhat arbitrary. Additionally, irrespective of the accuracy of the cut-off points separating lineages, any genetic-based taxonomy may struggle with the noise generated by recurrent and backwards mutations [6, 15], as well as lineage extinction, both prevailing properties of SARS-CoV-2 evolution (Fig. 4A and C).

## SARS-CoV-2 GENOTYPE AND PHENOTYPE

While most mutations are expected to have no functional impact [163], some sites in the SARS-CoV-2 genome have been associated with changes in viral phenotype. Unsupervised assessment of the phenotypic effect of mutations has focused on characterizing proximal phenotypes such as binding affinity to the ACE2 host cell receptor or spike protein expression revealing a heterogenous phenotypic landscape over the spike protein [164]. Such an approach pinpointed spike mutation N501Y, characteristic of three of the five currently identified VoCs, as having a notable effect on receptor binding [164, 165]. Functional evidence for association of individual mutations with traits such as receptor binding affinity or gene expression provides useful mechanistic insight. However, more complex traits, such as virulence, transmissibility and immune escape, are more relevant epidemiologically. The genetic basis of complex traits is more challenging to infer mainly because they are affected by multiple mutations that do not necessarily act independently of each other. As such, a specific mutation may not always lead to the same phenotypic changes in different SARS-CoV-2 genetic backgrounds.

There is indeed evidence for extensive epistasis being involved in the evolution of SARS-CoV-2, with the fitness of viral lineages likely dependent on combinations of co-adapted mutations and deletions [166, 167]. More transmissible SARS-CoV-2 lineages did not primarily evolve through the sequential accumulation of mutations [15]. Instead, some VoCs seem to have emerged through the rapid acquisition of a set of mutations and deletions rarely observed together previously. For Alpha and Omicron in particular, it is suspected that new combinations of mutations arose during long-term infection in immunocompromised hosts [168, 169]. Rapid accumulation of rare mutations has been observed in chronic infections [170] and persistent infection in immunocompromised hosts may provide the virus with a possibility to explore a wider range of the fitness landscape. Favourable combinations of mutations that are deleterious in isolation may be unlikely to be acquired sequentially during transmission among immunocompetent hosts, as those less fit fitness lineages may rapidly go extinct. Persistent infections in immunocompromised hosts may allow such transient low fitness lineages to persist, with some eventually acquiring novel combinations of well co-adapted mutations. The evidence for the emergence in an immunocompromised host is particularly strong for the Omicron VoC, which acquired a large number of non-synonymous mutations in the immunodominant regions of the spike RBD region [22]. Additionally, Omicron-associated mutations confer significant escape from neutralizing antibodies but not T cell immunity [171, 172]. The unusual mutation pattern of Omicron might have arisen in an untreated HIV/AIDS patient with advanced CD4+ T-cell lymphopenia, who was unable to clear the infection [173]. Interestingly, Omicron comprises two deeply diverging lineages, BA.1 and BA.2 (nextstrain clades 21K and 21L). It is possible that both emerged in the same host before independently spreading in the community.

Recombination is also key for viral evolution as it allows for the combination, within a single genome, of mutations that arose independently in different genetic backgrounds. It is generally accepted that the emergence of all three human epidemic coronaviruses: SARS-CoV-1 [174–176], MERS-CoV [177] and SARS-CoV-2 [1, 178, 179] involved recombination events. Recombination also provides a mechanism for the generation of new antigenic combinations within species [180]. This can occur through the exchange of discrete segments of genetic material of different origins, well exemplified by antigenic shift in influenza [181, 182]. Recombination, however, requires co-infection of the same host by distinct lineages [183]. So far, relatively few recombinant SARS-CoV-2 genomes have been identified [184–188]. The relatively small number of recombinant SARS-CoV-2 genomes detected to date is intriguing given the large number of genomes available and epidemiological settings likely favouring co-infections by distinct lineages, for example, during mid 2021 (when prevalence was high and Alpha and Delta VoC were in co-circulation in multiple regions of the world). This may in part stem from difficulty in identifying recombinants because of low levels of SARS-CoV-2 genetic diversity in circulation at this stage. Low levels of genetic recombination in SARS-CoV-2 would however be in variance with the high recombination rates reported for other coronaviruses, including in human [116, 189–192] and animal-associated lineages [193–195], as inferred from genomic approaches [196], observed in cell culture [197, 198] and *in vivo* [199]. The low proportion of recombinant genomes detected to date might also in part be attributable to most of them having lower fitness, with recombinant lineages becoming extinct before they can be sampled.

Any replicating entity will be present in the future at a higher frequency if it makes more copies of itself [200]. Viruses are no exception to this fundamental rule of evolution. As such, through whatever means, viral lineages will evolve towards higher possible transmissibility. Efforts to computationally assess and predict transmissibility have focused on approaches based on logistic regression models of lineage growth rates [201] or phylogeny-based quantification of the association between carriage of specific mutations leading to an increase or decrease in the number of inferred descendants [150]. The latter approach capitalizes on the high rate of recurrent substitutions and deletions in SARS-CoV-2 [6, 15, 150, 202, 203] (Fig. 4A insets). Both those approaches to estimate transmissibility (viral fitness) highlight the propensity for a plethora of mutations, not only those within the critical spike RBD, to be implicated in increasing viral fitness [150, 201]. This is not entirely surprising since transmissibility reflects multiple underlying mechanisms such as the binding affinity to the human receptor ACE2 (i.e. fewer viral particles needed to generate a new infection) [164, 204], or viral replication rates [205, 206], or the ability to (re-)infect a larger fraction of the human population through immune escape.

Genetic determinants of virulence are difficult to assess not least because virulence is not a property of a pathogenic strain alone, but rather of an interaction with its host. Moreover, there are currently no large datasets available with the necessary information to associate individual viral mutations with disease severity and outcomes, while controlling for key host factors such as age, sex or health. It is sometimes assumed that as a pathogen adapts to its host, it becomes intrinsically less virulent [207, 208]. However, such a prediction is only true under two conditions. The first is when transmission is primarily vertical (e.g. from mother to child) as in human cytomegalovirus where fitness of the virus and the host are closely correlated, so that the pathogen would reduce its transmissibility by harming its host. The second is for extremely lethal pathogens where mortality of the host upon infection is so high that it precludes transmission of the pathogen. The textbook example for pathogen attenuation is myxomatosis in rabbit populations with initial mortalities of 99% upon initial introduction of the pathogen going down after a few years of coevolution [209]. Neither of those conditions are fulfilled by SARS-CoV-2. Despite its mortality and morbidity burden, SARS-CoV-2 remains a moderately lethal pathogen. Moreover, about half of transmissions are from pre- and asymptomatic carriers [210], which further reduce the selective pressure on the virus to spare its host.

The morbidity and mortality resulting from SARS-CoV-2 infection have however reduced significantly since the start of the pandemic and will do so further as an increasing proportion of the human population is immunized through natural infection and vaccination. However, the evolution of the intrinsic virulence of SARS-CoV-2 is difficult to predict. The Alpha and Delta variants were both associated with increased virulence, with a higher proportion of people infected requiring hospitalization [18, 211–214]. The higher virulence of the Alpha and Delta VoCs is likely a secondary (pleiotropic) consequence of their increased replication rate and transmissibility. Conversely, all major Omicron lineages in circulation at the time of writing (BA.1, BA.2, BA.4 and BA.5) are intrinsically less virulent than the Alpha and Delta VoCs [215] and their severity falls below that of early pandemic strains. One explanation for Omicron’s reduced pathogenicity has been its altered use of TMPRSS2. In particular, it has been suggested that the Omicron spike protein tends to use the alternative cathepsin-mediated activation rather than the canonical TMPRSS2 pathway [216–218]. This property simultaneously results in a shift in tissue tropism away from TMPRSS2 expressing cells (common to the lungs) to the upper respiratory tract, resulting in lower intrinsic virulence [217, 218].

A lower frequency of severe COVID-19 and fatalities from Omicron infection appears to be the result of both intrinsic differences in the virus pathogenicity and widespread cross-reactive immunity [219]. This is mirrored in vaccine breakthrough data from South Africa that shows reduced frequency of severe disease with Omicron relative to previous VoCs [220]. Relatively low case fatality rates were recorded during Omicron waves both in countries with high vaccination rates (e.g. New Zealand) and high levels of prior infection (e.g. South Africa). Very high levels of neutralizing antibodies may be able to overcome partial escape to provide some cross-neutralization, while non-neutralizing immunity through antibody-dependent cellular cytotoxicity and T cells is postulated to provide protection against disease when breakthrough Omicron infection occurs [221–224]. However, in populations with hardly any prior infection and low immunization rates among the elderly, Omicron still caused devastating outbreaks as was observed in Hong Kong, which recorded the highest per capita death rate of any country, at any time throughout the pandemic in March 2022.

## HOW ‘SPECIFIC’ IS SARS-CoV-2-SPECIFIC IMMUNITY

A major focus of SARS-CoV-2 research has been to understand the extent to which immunity generated by infection or vaccination can cross-recognize and cross-protect against different lineages of SARS-CoV-2 and other members of the Coronaviridae family. This is particularly relevant now that the global population consists of a mixture of individuals with different histories of exposure to SARS-CoV-2 antigens [225].

The majority of immunocompetent individuals infected with SARS-CoV-2 develop both T and B cell responses and these remain detectable in the circulation for at least 9–12 months [226, 227]. Previous infection has been shown to offer protection from reinfection with the same SARS-CoV-2 variant for at least 6 months [228–230], although this may be reduced in the elderly, or when exposed to a heterologous viral strain. Likewise, vaccine-induced immunity against SARS-CoV-2 has shown impressive efficacy against severe disease, hospitalization and death, at least in the short term [231]. Accumulating data suggest that T cells play an important role in the resolution of SARS-CoV-2 infection [232, 233] and probably against disease following breakthrough infection post-vaccination. However, it is likely that vaccine efficacy against infection is mediated predominantly by antibody responses. For example, titres of binding antibodies to spike and RBD are predictive of breakthrough Delta infection following mRNA-1273 vaccination, supporting their utility as correlates of protection (COP) [231].

Waning of effective immune protection over time can occur through a combination of changes in the circulating virus itself and subsequent loss of recognition by the immune response (escape), and/or by reduction in the number of immune effectors over time, such as antibody levels (reflective of plasma cells), memory B cells (able to generate new affinity-matured antibodies upon re-infection) and memory T cells. SARS-CoV-2 binding and neutralizing antibodies show measurable waning post-infection [226, 234, 235], which may correlate with risk of breakthrough infection [236]. However, memory B cells can persist after SARS-CoV-2 infection and vaccination [226, 237] even after neutralizing antibodies become undetectable in the blood [238].

Many mechanisms can be used by viruses to evade adaptive immunity (e.g. shielding epitopes with glycans/lipoproteins, cell–cell spread, induction of interfering antibodies and disrupting antigen presentation). For SARS-CoV-2, much attention has been paid to mutational changes in the spike protein [239]. Point mutations within the spike can lead to loss of recognition by monoclonal antibodies, including those used as therapeutics [164, 240], however, single mutations do not significantly evade polyclonal responses (that recognize many regions of spike) present in convalescent serum. Combinations of these point mutations are, however, common to certain variants and when occurring together result in a significant reduction in the ability of antibody responses to recognize and neutralize variants. This has been well demonstrated by the emergence of the Omicron BA.1 variant with 30 spike substitutions, 6 deletions and 3 insertions, which together lead to significantly reduced neutralization *in vitro* by convalescent and post-vaccination serum relative to other strains [223, 224, 241] and a higher rate of breakthrough infection.

Prior to the emergence of the Omicron variant, the rise and decline of VoCs did not broadly correlate with immune escape. Alpha emerged with a comparatively small number of spike mutations but was relatively well neutralized by convalescent and post-vaccination serum [242]. The Beta and Gamma variants demonstrated much more evidence of antibody escape, for instance evading 12 of 17 monoclonal tested and convalescent and post-vaccination serum [166, 242, 243], however, they failed to outcompete the Alpha VoC that was globally dominant at the time. Sufficient cross-reactive immunity may have been retained despite the changes in Beta and Gamma spike proteins [225]. Subsequently, Delta outcompeted all other strains, accounting for up to 99% of infections globally in October 2021 despite showing modest evidence of immune escape relative to ancestral strains [244–246], which suggests that infectivity and transmissibility may have been the dominant factors determining VoC in the earlier stages of the pandemic. For example, S: E484K is the individual mutation most strongly implicated in partial immune escape yet the Beta and Gamma VOCs, each carrying E484K, failed to dominate in the same manner as Alpha, Delta and Omicron. The E484K mutation has also been acquired repeatedly by strains belonging to the Alpha and Delta VoCs (referred to as ‘Alpha+’ and ‘Delta+’) and significantly reduces neutralization in those genetic backgrounds [247, 248]. Yet, Alpha+ and Delta+ lineages have not been particularly successful. With a large proportion of the human population having been vaccinated and/or previously exposed to SARS-CoV-2 from early 2022, the major selective pressure likely shifted from higher infectiousness to an increased ability to (re-)infect already immunized hosts. The Omicron strains broke pre-conceptions of how much room SARS-CoV-2 spike had to evolve; Omicron spike contains a complex pattern of mutations that retain RBD ACE2 binding while changing the shape of the RBD sufficiently to escape a significant proportion of neutralizing antibodies [241].

As with antibody recognition, mutations within epitopes can also lead to escape from the cellular arm of the adaptive immune response, whereby T cells fail to recognize infected cells, for instance due to the mutant epitope failing to be processed and presented effectively, or due to loss of recognition of the MHC-peptide complex by the T cells receptor (TCR) [91, 249–252]. Studies to date suggest the majority of T cells induced by vaccination or infection retain their ability to recognize epitopes within Omicron and other variants [253, 254]. T cell responses to SARS-CoV-2 post-infection are highly multispecific, targeting multiple potential human leukocyte antigens (HLA)-restricted epitopes within all viral proteins, not just the structural proteins and surface epitopes available to antibodies [225, 255–258]. Notably, all widely used SARS-CoV-2 vaccines also induced T cells responses which are polyclonal and multispecific in most individuals, however, these are restricted to the proteins contained within the vaccine, currently largely the spike [231, 259–261]. HLA have evolved to be highly polymorphic, meaning that the epitopes recognized vary substantially between individuals, implying that T cell escape variants are less likely to contribute to transmission within a population unless representing a highly immunodominant response restricted by a frequent HLA allele. TCRs, the receptors by which infected cells are recognized, are also randomly generated meaning each individual recognizes epitopes with a unique combination of TCRs; therefore, viral mutations will impact recognition in subtly different ways for each individual. Overall, these mechanisms reduce the selection pressure for individual escape mutations at the population level, in particular for an acute resolving infection. In other words, the advantage gained by mutating a single epitope would only be advantageous in another host if they share the same HLA restriction.

Related to SARS-CoV-2 lineage cross-immunity, pre-exposure to non-SARS-CoV-2 viruses can mediate cross-immunity. For instance, T cell responses that can cross-recognize SARS-CoV-2 can be detected in pre-pandemic samples taken before it circulated in humans in as many as 80% of individuals, depending on the sensitivity of the assay used [227, 262–265]. Due to sequence conservation with SARS-CoV-2, many groups have started to investigate the possibility that these pre-existing T cells were induced by universal exposure to HCoVs [256, 257, 262, 266, 267]. While it is unlikely that HCoVs are the sole source of pre-existing T cell responses [30], it has been demonstrated that T cells can cross-recognize SARS-CoV-2 and all four HCoVs *in vitro* at certain epitopes [256, 262, 266–268] and that T cells transduced with TCRs from convalescent samples could recognize HCoVs and SARS-CoV-2 with similar affinities [269].

Prior immunity would be expected to limit disease on exposure to SARS-CoV-2 as pre-existing memory T cells are imbued with characteristics which make them more efficient at viral control than naïve T cells. As an example, pre-existing T cells that target the highly conserved core replication transcription complex (NSP7 polymerase cofactor, NSP12 polymerase and NSP13 helicase) were shown to be enriched in health care workers that showed signs of exposure to SARS-CoV-2 but who appeared to control infection before it was detectable by PCR or induced an antibody response [262]. This early control of viral replication, before an infection could be established, may be explained by the rapid response of pre-existing T cells that target the earliest expressed proteins of the viral lifecycle in ORF1ab. Pre-existing cross-reactive T cells expand *in vivo* upon infection and vaccination [266] and have been associated with protection from infection [262, 270] and severe disease [271], but epidemiological data are still limited to support [272] or refute [273] that recent HCoV infection and associated immune responses are directly associated with protection from severe COVID-19.

Cross-reactive antibodies are less common in pre-pandemic samples, likely due to their targeting of less conserved structural viral proteins and greater sensitivity to point mutations altering structural epitopes [274, 275] and they have not been associated with protection from disease [276]. Nonetheless, it has been suggested that pre-existing cross-reactive immunity is the reason we have so few endemic coronaviruses, rather than lack of opportunity or lack of identification of coronavirus outbreaks in humans [277]. For another coronavirus to establish itself in the human population like SARS-CoV-2 did, it would have to circumvent significant pre-existing immunity generated by previous coronaviruses in circulation.

The biggest unknown in SARS-CoV-2 immunity is what correlates with protection from disease and from infection. Attempts have been made to identify antibody COP for SARS-CoV-2 by integrating efficacy and antibody data from vaccine trials [278, 279]; however, a single COP may be hard to identify for all SARS-CoV-2 VoCs without measuring variant-specific neutralizing antibodies, as well as non-neutralizing antibodies, and cellular immunity. A greater understanding of the immune responses at mucosal airway surfaces, the site of viral control, will likely be required [280]. Despite difficulties in accessing samples, compartmentalized immunity within the airway mucosa in the form of tissue-resident T cells [281–283] and local IgA, memory B cells [284, 285] have been described and associated with protection from severe disease and infection with SARS-CoV-2 [286].

A growing body of evidence suggests that vaccines for global health priorities should be focused on inducing both antibodies and T cells [287, 288]. Due to their longevity, multispecificity and propensity to target conserved regions, T cells could be particularly effective at mediating long-term protection against SARS-CoV-2. Since most T cell targets lie outside of the spike protein [255, 256, 262], it may be prudent to include non-spike antigens in vaccines, possibly targeting regions that are conserved across the wider *Coronaviridae*. Clinical trials are already underway to test second-generation vaccines to improve durability, infection and transmission blocking and the potential for inducing cross-reactive immunity that can protect against future SARS-CoV-2 variants and acquisition of novel coronaviruses in the future.

## THE FOUR FUNDAMENTAL FORCES OF AN EPIDEMIC

**Figure 5:**
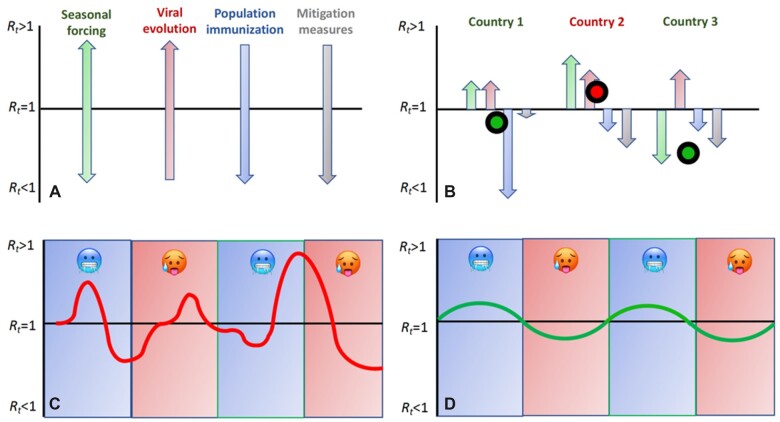
Schematic representation of the four main forces acting on pathogen transmission. *Notes*: (**A**) Effect of the four main forces on transmission. (**B**) Cumulative effect of the forces in three hypothetical countries. In countries 1 and 3, the cumulative effects of the four forces keep the epidemic from growing (*R *<* *1), whereas in country 2, the epidemic is growing. (**C**) Epidemic behaviour over a 2-year period under a continental climate with blue area depicting colder months in the year and red warmer months. (**D**) Seasonal dynamic of endemic pathogen with smaller amplitude of cases and regular peaks during the colder months.

The epidemic dynamic of respiratory viruses such as SARS-CoV-2 is driven by four main factors, namely, seasonality, viral evolution, population immunization rates and mitigation measures affecting host behaviour (Fig. 5A). Seasonal forcing expresses the higher transmissibility of respiratory viruses during winter than summer in both hemispheres. This is in part due to physical conditions, such as viruses remaining infectious for longer in cold, dry air [289] and under low sunlight (UV) exposure [290]. However, there is also a host behaviour component with humans tending to spend more time indoors in poorly ventilated conditions during the winter [291, 292]. Viral evolution will always tend to push transmissibility upwards but it is expected to tend towards a fitness maximum. Population immunization reduces viral transmissibility by removing susceptible hosts from the population, but is constantly refuelled by viral antigenic drift, waning of immunization and introduction into the population of unexposed newborn hosts, thus eventually reaching a dynamic quasi-equilibrium. Finally, mitigation measures, which encompass interventions such as aiming to increase social distancing, will reduce transmission but for the most part are unlikely to stay in place indefinitely.

Together these four forces will jointly affect the effective reproduction number of a pathogen (*R_t_*), the average number of new infections caused by a single infected individual at time *t*. When *R_t_* is above 1.0, case numbers increase and when it falls below 1.0, they go down (Fig. 5B). In an epidemic dynamic, *R_t_*, and hence case numbers, can fluctuate wildly leading to marked epidemic waves (Fig. 5C). Herd immunity is reached when *R_t_* falls below 1.0, but for viruses such as SARS-CoV-2, where immunization against re-infection is relatively short lived, this endemic state is only transient. The only force in the system that remains unaltered is seasonal forcing, which ultimately becomes a major driver of the system, leading to higher case numbers in winter than in summer in the temperate zone (Fig. 5D).

Essentially all ∼200 endemic human respiratory viruses are seasonal in temperate regions of the world. Many endemic respiratory viruses including the four endemic HCoVs also tend to exhibit a biannual dynamic, with case number higher every second year [293]. This might in part stem from a non-equilibrium state caused by immune protection from reinfection following prior exposure lasting on average for longer than 1 year, but less than 2. SARS-CoV-2 is no exception to the general pattern of seasonality, and its transmissibility, at the time of writing, is already higher in winter. Seasonal variation in the transmissibility of SARS-CoV-2 had little impact during the early stages of the pandemic. This is because as long as immunization rates remained limited, SARS-CoV-2 could readily transmit at any time of year as long as conditions were otherwise favourable, and case rates were mainly driven by variation in the stringency of mitigation measures and the emergence of more transmissible lineages. For instance, India experienced a major epidemic peak outside the winter season, during the spread of the Delta variant in late spring 2021. As immunization levels increase further, SARS-CoV-2 is expected to adopt a seasonal endemic dynamic, with outbreaks and epidemics mostly happening in winter.

## THE GOOD, THE BAD AND THE UGLY

While there is little doubt that SARS-CoV-2 will eventually become a seasonal endemic pathogen, there is limited consensus in the scientific community about what ‘endemic SARS-CoV-2’ entails in terms of future morbidity and mortality, both upon infection and through long COVID/PASC (post acute sequelae of SARS-CoV-2 infection). However, during the endemic phase of COVID-19, morbidity and mortality will primarily be dictated by the ability of SARS-CoV-2 to bypass global host immunity and the intrinsic virulence of future SARS-CoV-2 variants.

Worst case scenarios include high case numbers and circulation of a diversity of serotypes with limited protection across strains, possibly fuelled by reverse anthroponosis and recombination between human and animal coronaviruses. For example, infectious bronchitis virus (IBV), a *Gammacoronavirus* infecting chicken, circulates in the form of multiple serotypes with highly divergent sequences in their spike protein and minimal cross-immunization between certain strains in circulation [294]. This would present a highly challenging situation, leading to constantly high case numbers and a need for continual vaccine updates to match at least a subset of dominant serotypes in circulation. While such a scenario of diversification into multiple serotypes cannot be formally ruled out as the future for endemic SARS-CoV-2, it remains relatively unlikely. Humans, as long-lived hosts, are typically exposed multiple times in their lives to the four HCoVs and will in all likelihood be to SARS-CoV-2 in the future. Repeated exposure to the five coronaviruses in circulation is expected to restrict the immunological space that SARS-CoV-2 lineages can explore at any time [277]. The extent to which immune-escape mutations in the spike RBD act independently from each other will be critical for the emergence of new serotypes through previously unexplored epistatic combinations. There is currently no consensus on the evolutionary potential of SARS-CoV-2 to generate new serotypes through novel epistatic combinations of mutations [295, 296]. However, at the least, we can anticipate that SARS-CoV-2 will undergo antigenic drift in common with other HCoVs [133].

A situation where SARS-CoV-2 will circulate in the future as one or two serotypes at any time, constantly evolving through antigenic drift and with sequential lineage replacement represents the most likely scenario. This is the situation we currently experience with the four HCoVs and seasonal influenza A and B. People are generally infected for the first time to both HCoVs and seasonal influenza early in life and build immunity through multiple exposures throughout life. The majority of influenza infections are asymptomatic and people tend to get exposed to influenza multiple times [297]; the same is true for HCoVs [127–129]. In terms of epidemiology and public health, one major difference between HCoVs and influenza is the higher virulence of seasonal influenza. HCoVs are generally considered to exert only a minor burden on human health, and are often tellingly categorized among ‘common colds’, even if they are not always harmless, in particularly in the elderly. While also often underascertained, the burden of influenza tends to be more widely recognized. A recent study estimated the number of yearly deaths associated with seasonal influenza to around 400 000 globally (with considerable year on year variation), with around two-thirds of these among people over the age of 65 years old [298]. Additionally, influenza A undergoes rare events of antigenic shifts, which have not been documented in HCoVs to date. An antigenic shift corresponds to the emergence of a new lineage, to which the population has no or limited immunity, and which leads to an influenza pandemic as in 1918, 1957, 1968 or 2009. The emergence of the Omicron variant could be considered as largely analogous to an influenza A antigenic shift. The extent to which the emergence of Omicron was an exceptional event or part of reoccurring future pattern will be determinant for the future dynamic of SARS-CoV-2.

COVID-19 has been the worst respiratory disease pandemic since the ‘Spanish flu’ in 1918/20 when influenza H1N1 may have killed up to 50 million. The 1918–20 flu pandemic came to an end with H1N1 becoming the agent of endemic seasonal flu until it got displaced by the 1957–58 H2N2 pandemic lineage. The 1918 H1N1 lineage then made a comeback in the 1970s, again contributing to the yearly burden of seasonal flu until it got likely displaced in humans by the 2009 pandemic H1N1pdm09 lineage [299]. While pandemics of respiratory viruses end, the causal agents remain in circulation for decades. As such, whether SARS-CoV-2 will in time become a fifth ‘common cold HCoV’, or exert a more significant burden on human health comparable to, or even higher than, seasonal influenza will largely depend on the intrinsic virulence of future viral lineages. We have essentially no control over the global evolution of the virus and the trajectory in terms of virulence of future SARS-CoV-2 variants is unknown, if not unknowable. However, what we have considerable control over is the morbidity and mortality associated with endemic SARS-CoV-2 in the future. By far the best tool is global vaccination of the elderly and those otherwise most at risk, which needs to be scaled up and maintained globally if we hope to live with a fifth HCoV, rather than an additional seasonal flu-type virus.
